# Integrated Approach to Diagnosing Limb‐Girdle Muscular Dystrophies in Resource‐Limited Settings

**DOI:** 10.1155/humu/1321391

**Published:** 2026-10-02

**Authors:** Hammad Yousaf, Lubaba Bintee Khalid, Affan Ahmed, Rafia Zafar Ghumman, Shaheer Hassan, Hijab Zahra, Ghazala Zafar, Kinza Rubab, Hazaratullah Azakai, Haq Nawaz Khan, Qamar Mahmood, Asmat Ali, Bilal Ahmad Mian, Zafar Ali, Mohammad Raza, Sanam Bano, Khairunnisa Mukhtiar, Uzma Abdullah, Shahnaz H. Ibrahim, Shahid M. Baig, Mathias Toft, Salman Kirmani, Zafar Iqbal, Ambrin Fatima

**Affiliations:** ^1^ Department of Biological and Biomedical Sciences The Aga Khan University Karachi Sindh Pakistan aku.edu; ^2^ Division of Women and Child Health The Aga Khan University Karachi Sindh Pakistan aku.edu; ^3^ Islam Medical College Sialkot Punjab Pakistan imdc.edu.pk; ^4^ Medical College The Aga Khan University Karachi Sindh Pakistan aku.edu; ^5^ Department of Medicine II University Hospital Würzburg Würzburg Germany ukw.de; ^6^ University Institute of Biochemistry and Biotechnology PMAS-Arid Agriculture University Rawalpindi Punjab Pakistan uaar.edu.pk; ^7^ National Institute of Rehabilitation Medicine (NIRM) Islamabad Islamabad Capital Territory Pakistan; ^8^ Department of Pathology and Laboratory Medicine The Aga Khan University Karachi Sindh Pakistan aku.edu; ^9^ Faculty of Rehabilitation and Allied Health Sciences Riphah International University Islamabad Islamabad Capital Territory Pakistan riphah.edu.pk; ^10^ Department of Biotechnology Genetics Forensic and Microbiology University of Swat Swat Khyber Pakhtunkhwa Pakistan uswat.edu.pk; ^11^ Department of Pediatrics and Child Health The Aga Khan University Hospital Karachi Sindh Pakistan aku.edu; ^12^ Faculty of Life Sciences Health Services Academy Islamabad Islamabad Capital Territory Pakistan hsa.edu.pk; ^13^ Institute of Clinical Medicine University of Oslo Oslo Norway uio.no; ^14^ Department of Neurology Oslo University Hospital Oslo Norway oslo-universitetssykehus.no; ^15^ Centre for Regenerative Medicine and Stem Cells Research The Aga Khan University Karachi Sindh Pakistan aku.edu

**Keywords:** *CAPN3*, *COL12A1*, *DYSF*, ES, *LAMA2*, LGMD, *SGCA*

## Abstract

Limb‐girdle muscular dystrophies (LGMD) are a genetically heterogeneous group of neuromuscular disorders characterized by overlapping clinical features, including progressive muscle weakness and wasting, elevated creatine kinase (CK) levels, and motor and skeletal abnormalities. Due to phenotypic overlap and variable severity, clinical diagnosis alone is often unreliable. Incorporation of genetic investigations to aid diagnosis/differential diagnosis has accelerated the diagnostic process but remains largely limited to developed countries. Here, we employed genetic testing as a first‐tier approach to simplify the diagnostic journey in a resource‐limited setting, combined with preliminary functional analyses to identify the underlying genetic cause. Our cohort included 46 affected individuals (31 males, 15 females) from 29 families with a mean age of 23.59 ± 11.87 years and a mean age at onset of 14.8 ± 9.2 years. All individuals presented with progressive muscle weakness, elevated CK levels, movement difficulties, and gait abnormalities. Genetic testing identified pathogenic/likely pathogenic variants in *CAPN3* (8 families)*, DYSF* (15 families), *LAMA2* (4 families), and *SGCA* (1 family), whereas a variant of uncertain significance was identified in *COL12A1* (1 family). Preliminary functional investigations in patient‐derived fibroblasts demonstrated that the COL12A1: p.(Val1051Leu) identified in Family 1 results in reduced mRNA and protein abundance. Overall, 25 unique variants were identified, including 17 novel variants. This study highlights the utility of integrating genetic and functional approaches to facilitate accurate diagnosis in low‐ and middle‐income settings.

## 1. Introduction

The term “limb girdle muscular dystrophy (LGMD)” was coined in 1954 by Walton and Nattrass [1] to describe a group of disorders exhibiting progressive muscular weakness and atrophy typically affecting proximal limb girdles. The estimated prevalence of LGMD is between 1/14,500 and 1/123,000 [2]. LGMD arise from defects in the genes encoding for structural, functional, and maintenance components of muscle fibers that localize to diverse cellular compartments, including the nucleus (e.g., transportin‐3), sarcomere (e.g., titin), sarcolemma (e.g., sarcoglycans and dysferlin), and extracellular matrix (e.g., collagen and laminin). Consequent to the functional effect of the disease‐causing variants, these disorders are inherited in an autosomal dominant (Type 1 LGMD or LGMDD) or autosomal recessive (Type 2 LGMD or LGMDR) manner [2–4] with recessive forms being more common and generally more severe [5].

Collectively, there are more than 30 types of LGMD that have been hitherto reported with distinct genetic determinants, variable disease severity, and overlapping clinical features. Disease severity depends on the onset of the disease (childhood vs. adolescence), beginning from the proximal muscles, but it progresses symmetrically to affect all muscle types and may lead to other organ anomalies like cardiomyopathy or respiratory failure [6]. LGMD2A/LGMDR1 is the most common subgroup that affects 40%–50% of all LGMD cases [7] and is caused by dysfunction of *CAPN3* (calpain‐3) (a muscle‐specific enzyme) [8]. It begins in the gluteal and hip adductor muscles [9] with variable disease onset and progression [10]. LGMD2B/LGMDR2 is caused by the deficiency of *DYSF* (involved in vesicular trafficking) [11]. Patients show no ambulation problems in childhood, but ultimately develop progressive muscle weakness without inflammation [12, 13]. Except for deficiency for *CAPN3* and *DYSF*, which are functional proteins to cause LGMD, the rest of the LGMD categories are caused by malfunctions of structural proteins. Sarcoglycan‐related LGMD (LGMD 2C–2F/LGMDR3 to R6‐sarcoglycanopathies) are next in prevalence and caused by proteins that form dystrophin‐associated protein complex (DAPC) to maintain muscle fiber integrity [2]. Sarcoglycanopathies, in general, initially affect pelvic girdle, followed by progression to pectoral girdle and scapular winging [5]. These are often associated with raised creatine kinase (CK) levels [2, 3]. Regardless of the underlying defects and incidence of these individual subtypes, the burden on the healthcare system and affected families is enormous. This is driven in part by the overlapping continuum of phenotypic outcomes with variable severity, genetic heterogeneity, and the lack of a cure, which together complicate diagnosis, delay targeted interventions, and increase long‐term care needs. This challenge is further exacerbated in low‐ and middle‐income countries (LMICs) such as Pakistan, where high consanguinity, limited infrastructure and expertise result in a substantial proportion of patients remaining without a definitive diagnosis.

Diagnosis solely based on clinical features is often prone to errors since phenotypic outcomes—driven by genotype—can be subtly influenced by factors such as environment, age, and genetic background. [14–17]. For diseases such as LGMD with predominantly established gene‐disease implications, the incorporation of genetic investigations to aid diagnosis/differential diagnosis has been accelerating the diagnostic process, enabling genetic counseling and timely palliative care [18–22]. To this end, we employed a comprehensive approach to investigate 46 affected individuals from 29 families presenting with the features of LGMD. We performed exome sequencing (ES), conducted segregation analysis through Sanger sequencing, and carried out functional analyses (in Family 1) to identify the underlying genetic cause of the disease in these families.

## 2. Material and Methods

### 2.1. Recruitment of Research Subjects

This study was approved by the institutional review board of the Aga Khan university vide letter no. 2021‐65141‐9346 and is conducted in line with the principles of Declaration of Helsinki. Families were recruited through community‐based sampling from different regions of Pakistan and from the neurology clinic at Aga Khan University Hospital, Karachi. All families were unrelated to each other. After written informed consent for participation and subsequent publication, peripheral blood was drawn from the proband (in all families) and available family members of the probands (community‐based sampling) preferably parents and siblings.

### 2.2. Molecular Genetic Investigations

Clinical genetic testing was performed for the subjects recruited from hospital clinics through commercial testing laboratories (mainly InterGen). Families that were recruited through community‐based sampling were processed in‐house. Genomic DNA was extracted using Quick‐DNA Miniprep Plus Kit (Cat# D4069) according to manufacturer′s protocols. SureSelect Human All Exome V6 (Agilent Technologies, Santa Clara, California, United States) was used to prepare NGS libraries which were run using NovaSeq 6000 (Illumina, Santa Clara, California, United States). Downstream bioinformatics pipeline and variant prioritization were performed as described [23, 24]. Functional annotation of the variants was carried out by ANNOtate VARiation (ANNOVAR) [25]. Variant filtering was performed by FILTUS [26]. Variants classification was based on the American College of Medical Genetics and Genomics (ACMG) guidelines [27]. In selected cases, segregation analysis of the candidate variants was performed by Sanger sequencing in all available individuals of the families. PCR primers and conditions used for variant verification are available on request.

### 2.3. Amino Acid Conservation Analysis

Amino acid sequence of translation IDs corresponding to the canonical transcripts of *COL12A1* orthologs in different species was obtained from Ensembl (https://www.ensembl.org/). We used Clustal Omega at EMBL‐EBI for alignments [28]. The translation IDs used were as follows: *Homo sapiens*: ENSP00000325146.8; *Pan troglodytes*: ENSPTRP00000031362.5; *Loxodonta africana*: ENSLAFP00000024738.1; *Equus caballus*: ENSECAP00000036924.1; *Mus musculus*: ENSMUSP00000071662.7; *Rattus norvegicus*: ENSRNOP00000075422.3; *Bos taurus*: ENSBTAP00000026725.6; *Felis catus*: ENSFCTP00005021746.1; *Oryctolagus cuniculus*: ENSOCUP00000013193.4.

### 2.4. Primary Skin Fibroblast Culture for *COL12A1* Assays

Skin biopsy from the patient with *COL12A1* homozygous variant (Family 1: IV:2), as well as from an age, gender, and ethnicity matched control was mechanically disassociated and cultured in multiple wells of 24‐well plate (Thermo Scientific, Cat# 144530), with each well containing 500 *μ*l of DMEM medium (Sigma‐Aldrich) supplemented with 10% fetal bovine serum (FBS) and 1% penicillin–streptomycin. Plates were placed at 37°C in 5% CO_2_ incubator. Biopsies were observed and media were regularly replenished until fibroblasts were derived.

### 2.5. Quantitative Real‐Time (qRT) PCR for *COL12A1* Relative Expression

Patient (Family 1: IV:2) and control cultured fibroblasts at comparable passage numbers, P8 and P10, respectively, were harvested in TRizol (Invitrogen) to extract total RNA using standard protocols. RNA was reverse transcribed using RevertAid First Strand cDNA Synthesis kit (Thermo Scientific Cat# K1622) according to the manufacturer′s protocols. qRT‐PCR was performed using SYBR green chemistry (Sigma‐Aldrich). 2^−*Δ* *Δ*Ct^ method was employed for estimating relative gene expression and data was normalized to *GAPDH* as the housekeeping gene. Each sample was run in triplicate. Primers are available on request.

### 2.6. Immunocytochemistry for *COL12A1*

For immunofluorescence staining of *COL12A1,* cultured fibroblasts were grown on cover slips for 3 days and were fixed using 4% paraformaldehyde. Cells were washed with PBS and permeabilized using 0.3% triton‐X, 1% PBS, and 1% bovine serum albumin (BSA) followed by blocking using same solution. Next, cells were incubated with COL12A1 primary antibody (Novus Biologicals COL12A1: Cat# NBP1‐88062, 1:100) overnight at 4°C. Next day, cells were washed thrice with PBS followed by secondary antibody treatment (Thermo Scientific anti‐Rabbit secondary antibody: Cat# A11034). After 1‐hour treatment with secondary antibody at room temperature, cells were washed and stained with DAPI for 5 min followed by fluorescent imaging.

### 2.7. *COL12A1* Immunoblotting

Whole cell lysate was obtained from patient and control cultured fibroblasts. Cells were harvested in RIPA lysis buffer (50 mM HEPES [pH 7.6], 1% Triton X‐100, 0.1% SDS, 50 mM NaCl, 0.5% sodium deoxycholate, 1 mM NaF, and 0.02 mM DTT) and incubated at 4°C for 1 h followed by centrifugation at 15000 RPM for 15 min. Supernatant was collected in a sterile 1.5‐mL tube. Lysate concentration was quantified using Pierce BCA Protein Assay Kit (Thermo Scientific Cat# 23225). We loaded 30 *μ*g of heat‐denatured cell lysate in RIPA buffer on a 10% polyacrylamide gel and resolved for 2.5 h at 90 V immersed in 1x Tris‐Glycine‐SDS running buffer (25 mM Tris, 192 mM glycine, 0.1% SDS) followed by overnight transfer to PVDF membrane in 1X Tris‐Glycine transfer buffer (25‐mM Tris base, 192 mM glycine, and 20% methanol) buffer. On the next day, the membrane was blocked with 5% BSA solution followed by primary antibody incubation (Novus Biologicals COL12A1: Cat# NBP1‐88062; COL6A2: Cat# H00001292‐M01; 1:1000 concentration in blocking solution; *β*‐actin: Cloud‐clone Corp Cat# CAB340Mi22, 1:1000) at 4°C overnight. The next morning, the membrane was washed thrice with TBST for 15 min each followed by secondary antibody (Goat anti‐Rabbit IgG‐HRP, Invitrogen Cat# 31460, 1:1000 [for *COL12A1*]; Goat anti‐Mouse IgG‐HRP, Invitrogen Cat# 31430, 1:1000 [for COL6A2 and *β*‐actin]) in blocking solution for 2 h at room temperature. Next, the membrane was TBST‐washed followed by visualization using SuperSignal West Pico PLUS Chemiluminescent Substrate (ThermoScientific) as per protocol on a ChemiDoc XRS imaging system (Bio‐Rad). Images were acquired under identical exposure conditions. Western blot band intensities were quantified using ImageJ (1.54p, NIH, Bethesda, Maryland, United States). Mean gray value (MGV) was measured for each band in a row using equal‐sized ROIs, with a same‐sized ROI used for background correction. Band intensity was inverted (255‐MGV) and background‐corrected by subtraction of background signal. Target protein (COL12A1 or COL6A1) was normalized to *β*‐actin from the same lane. Relative expression was calculated as the ratio of patient to control, with control set to 1.

## 3. Results

### 3.1. Biallelic Variants Are Identified in LGMD‐Related Genes in Unresolved Families With Muscular Dystrophy

The current study investigated 29 families affected by muscular dystrophies to establish a precise diagnosis through integrated genetic, clinical, and experimental investigations (Figure 1). The cohort comprised 46 affected individuals (31 males and 15 females), with mean ± standard deviation age of ranging 23.59 ± 11.87 years (range: 0.9–45 years; median: 20.5 years). The mean age at disease onset was 14.8 ± 9.2 years (Table S1). Eight families comprising multiple affected individuals (available for evaluation and genetic testing) were sampled through a community‐based sampling model, whereas the rest of the 21 families (probands only) were recruited from neurology and clinical genetic clinics at the Aga Khan University Hospital, Karachi, Pakistan.

**Figure 1:**
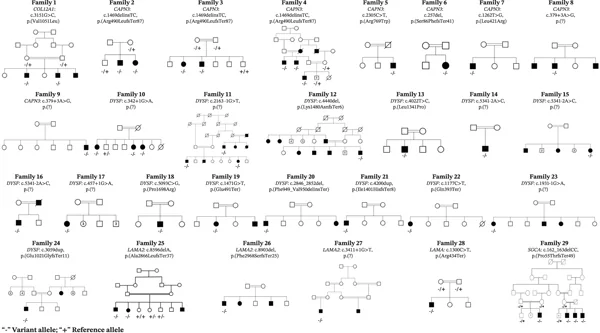
Pedigrees of the investigated families with limb‐girdle muscular dystrophies. Genotypes of the tested individuals are written below. −: variant allele; +: reference allele.

Clinically, all affected individuals exhibited a signature muscular dystrophy overlapping phenotype. Among 46 individuals, raised CK was observed in 45 (NA = 1), indicating near‐universal hyperCKemia. Lower limb weakness (44/44; NA = 2) and hip girdle weakness (42/42; NA = 4) were highly prevalent, accompanied by movement difficulties such as running and climbing stairs etc (41/41; NA = 5) and gait disturbances (40/41; NA = 5), reflecting predominant proximal muscle involvement. Standing difficulty was reported in 39/40 individuals (NA = 6), whereas upper limb weakness (38/44; NA = 2) and shoulder girdle weakness (35/39; NA = 7) were also common. Hypotonia was present in 33 individuals. Distal sparing was suggested by hands/fingers spared in 25 individuals, although decreased grip strength was noted in 24. Less frequent clinical findings included joint laxity and developmental delay. Structural musculoskeletal complications such as muscular contractures and spinal curvature anomalies were uncommon as well (Figure 2 and Table S1). Overall, the cohort demonstrates a clinical pattern characterized by markedly elevated CK levels, predominant proximal muscle weakness—particularly affecting lower limbs and hip girdle— and progressive motor impairment, whereas distal muscle involvement and skeletal deformities were relatively less frequent.

**Figure 2:**
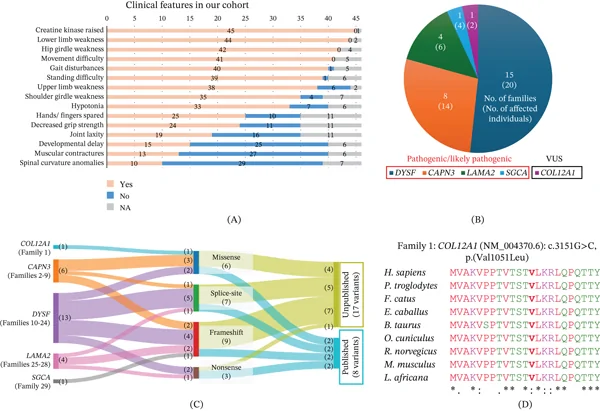
Clinical and genetic characteristics of the cohort with muscular dystrophies. (A) Frequency of key clinical features observed in the study cohort, bars represent the number of individuals with *yes*, *no*, or *not available (NA)* responses for each feature. (B) Distribution of genes and variant classification (according to ACMG criteria) identified as the plausible underlying cause of the disease in the cohort. Labels inside the chart: Top: No. of families, Bottom: No. of affected individuals. (C) Sankey diagram illustrating distribution of genes, numbers, and types of unique identified variants, and their previous publication status. Numbers inside the parentheses represent the count of unique variants. (D) Conservation analysis of the *COL12A1* across multiple species, demonstrating evolutionary conservation of the affected residue (Val1051) and flanking amino acids.

Fifteen families were investigated thorough ES (and subsequent targeted sequencing) and 14 were resolved through gene panel testing. Given the limited resources, ES was performed on one affected individual in most cases (except Families 1, 4, 11, and 25) followed by targeted testing of the identified variants in available family members. Genetic investigations revealed diagnostic findings in 28 families (*n* = 44 cases). Of these, affected individuals in eight families (*n* = 14 cases) harbor pathogenic/likely pathogenic variants in *CAPN3*, followed by 15 families (*n* = 20 cases) in *DYSF*, four families (*n* = 6 cases) in *LAMA2*, and one family (*n* = 4 cases) in *SGCA.* One family (*n* = 2 cases) harbored a variant of unknown significance (VUS) in *COL12A1* (Figure 2 and Table S1). In our cohort, we identified 25 unique variants across five genes, including *CAPN3* (6 variants), *DYSF* (13 variants), *LAMA2* (4 variants), *SGCA* (1 variant), and *COL12A1* (1 variant). Overall, 8 variants (30.8%) were previously published, whereas 17 (69.2%) were previously unpublished in medical literature. By variant class, frameshift variants were the most frequent (*n* = 9), of which 7 were unpublished. Splice‐site variants accounted for seven variants (five unpublished), missense variants for six (four unpublished), and nonsense variants for three (one unpublished). These findings highlight a substantial proportion of previously unreported variants, particularly truncating and splice‐site changes, within this cohort (Figure 2 and Table S1).

### 3.2. Biallelic Missense Variant in *COL12A1* Cause Ullrich Congenital Muscular Dystrophy 2 in Family 1

We recruited Family 1 from a rural area of Khyber Pakhtunkhwa province of Pakistan. Born at term to a healthy consanguineous couple, following a normal vaginal delivery (NVD), there are two male patients (IV:2 and IV:4) of 11 and 7 years, respectively (Figure 1). Gestational and birth history of both individuals were unremarkable. Individual IV:2 had 2.5 kg weight at the time of birth, whereas IV:4 had 2.7 kg birth weight. Both individuals presented with profound congenital hypotonia with early onset at 9 and 8 months, respectively, progressive generalized motor dysfunction, proximal muscle weakness involving both the shoulder and pelvic girdles, distal joint hyperlaxity, contractures in lower limbs, and truncal instability. In addition, facial and skeletal features in both individuals included swollen floppy face, with kyphosis, a high arched palate and hyperextensible pale skin with mild blue sclera. Furthermore, cognitive function was found to be normal in both patients. Only serum CK levels were found to be raised in both patients (Table S1).

We performed ES on both affected individuals and identified a novel homozygous missense variant in *COL12A1* (NM_004370.6): c.3151G>C, p.(Val1051Leu) as the most likely cause of the disease, as no other pathogenic or likely pathogenic variants were detected. Sanger sequencing validated the variant and confirmed segregation in recessive paradigm (Figure S1). Given the phenotypic overlap of patients (IV:2 and IV:4) with clinical features associated with *COL12A1*‐related autosomal recessive Ullrich Congenital Muscular Dystrophy 2 (MIM# 616470), this variant was considered as a candidate underlying the disease. The variant was classified as VUS as per ACMG guidelines [27]. As this was a missense variant, we next assessed the evolutionary conservation of Val1051 across multiple species. The analysis demonstrated that not only position 1051, but the entire surrounding amino acid region, is highly conserved across multiple species (Figure 2).

To assess the functional consequences of the *COL12A1*: c.3151G>C, p.(Val1051Leu), we first performed RT‐qPCR of RNA extracted from patient‐derived fibroblasts (Family 1: IV:2) to estimate *COL12A1* mRNA levels. Compared with controls, our data show a >50% reduction in *COL12A1* mRNA levels (Figure 3). Next, to confirm this finding, we performed immunocytochemistry using *COL12A1* specific antibodies. Fluorescent microscopy images showed a reduced expression of *COL12A1* protein within the cells of affected individual (IV:2) compared with controls (Figure 3). Furthermore, to evaluate impacts of the identified variant on total COL12A1 protein and to investigate, if any, potential impacts or cross influence on COL6A2, we performed immunoblotting on the lysate harvested from IV:2 derived fibroblasts. Blot analysis through MGV estimation revealed >80% reduction in COL12A1 levels in patient cells compared with controls, whereas the COL6A2 levels remained unaltered (Figure 3). Taken together, our qualitative data show that the *COL12A1* (NM_004370.6): c.3151G>C, p.(Val1051Leu) variant affects both mRNA as well as protein abundance in patient derived fibroblasts compared with the controls and represents the most plausible cause of the disease in these patients. However, these experimental observations are based on a single patient‐derived cell line, and the findings are derived from single representative experiments for each assay without quantitative assessment. Accordingly, these results provide preliminary insights and should be interpreted with appropriate caution.

**Figure 3:**
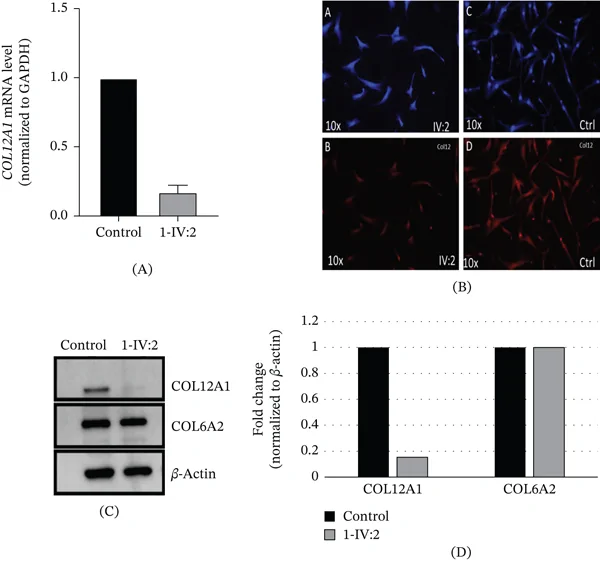
Functional characterization of the *COL12A1* variant. (A) Quantitative RT‐PCR analysis showing reduced *COL12A1* mRNA expression in fibroblasts derived from the affected individual (1‐IV:2) compared with control, normalized to *GAPDH*. (B) Immunofluorescence staining of cultured fibroblasts demonstrating altered COL12A1 expression in (A,B) patient cells compared with (C,D) controls. Upper panels show nuclear staining (blue), and lower panels show COL12A1 staining (red). Images were acquired at 10x magnification. (C) Western blot analysis of COL12A1 and COL6A2 protein abundance in A‐IV:2 as well as control cells, with *β*‐actin used as a loading control. (D) Mean gray value quantification of Western blot signals indicating a marked reduction of COL12A1 protein in the patient sample, whereas COL6A2 levels remain comparable to control.

### 3.3. Eight Families Harbor Variants in *CAPN3* Causing Limb‐Girdle Muscular Dystrophy Recessive 1

Family 2 was ascertained from the Rawalpindi district in Punjab province. Two affected individuals (II:2 and II:3) were born at term to unrelated parents through NVD and exhibited normal early development (Figure 1). At the time of the last evaluation, individual II:2 (male) was 44 years old, 160 cm tall, with a head circumference (HC) of 54 cm. He is unable to walk but can stand and sit with support. However, around school‐going age, he began to show developmental regression, characterized by recurrent falls and progressive muscle weakness with bilateral limb contractures. He walked with assistance until becoming nonambulatory at 21 years of age. His affected sister, individual II:3, was 41 years old, 150 cm tall, and had an HC of 54 cm at her most recent assessment. Like her brother, she experienced developmental regression beginning at a similar age, presenting with recurrent falls and progressive weakness leading to loss of ambulation and inability to stand; however, she can sit with support. Additionally, she has a history of seizures accompanied by foaming and has a palpable lump on the thigh near the hip joint. Both siblings had absent deep tendon reflexes, normal social interaction, and no history of recurrent infections (Table S1).

Family 3 belongs to Pukhtoon ethnicity. Two affected individuals (II:2‐Female and II:3‐Male) were 28 and 18 years of age at the time of recruitment (Figure 1). Patients presented a progressive form of muscular dystrophy and muscle weakness involving both proximal and distal muscles beginning around 18 years in II:2 and 15 years in II:3, leading to complete dependence for movement and feeding. Both individuals initially achieved all developmental milestones but developed severe muscle degeneration and weakness by late teenage years leading to nonambulation. Individual II:2 exhibited a more severe course, becoming bedridden soon after onset (Table S1).

Family 4 is an extended consanguineous kindred forming two interconnected loops, within which three individuals (IV:1, IV:2, and V:1) presented with overlapping neuromuscular and neurological manifestations (Figure 1). Despite variation in severity, all affected individuals demonstrated a broadly similar core phenotype characterized by adolescent onset (~19 years), progressive course, and symmetric proximal limb girdle weakness (Table S1). All individuals retained the ability to self‐feed, perform basic self‐care, and communicate verbally with preserved hearing. In addition to the shared clinical features, Individual IV:1 exhibited visual impairment, but no history of seizures or intellectual dysfunction at the time of last evaluation at 45 years of age. Individual IV:2 (40 years) had strabismus and positive oculomotor reflex abnormalities.

ES identified a pathogenic homozygous *CAPN3* variant (NM_000070.3): c.1469delinsTC, p.(Arg490LeufsTer87) [hg38: chr15‐42401755‐G‐TC] in affected individuals of Family 2, 3, and 4 segregating with the disease in a recessive paradigm (Table S1 and Figure S1).

In Family 6, the proband (II:2, female, 20 years old) presented progressive weakness involving both the pelvic and shoulder girdles. This was accompanied by hypotonia, contractures, lumbar lordosis, and markedly elevated CK levels. Muscle biopsy demonstrated degenerative and regenerative changes consistent with a dystrophic process. She experienced muscular weakness starting when she was 9 years old. By 13 years of age, she had developed difficulty climbing stairs, and an abnormal gait had become evident. She discontinued formal schooling in Grade 9 due to physical disability and subsequently continued her education through homeschooling. By the age of 17 years, she developed progressive upper limb weakness as well. There was no history of diurnal variation, bulbar symptoms, visual complaints, or scapular winging. Neurological examination showed reduced power in both upper and lower limbs, with proximal muscles more affected than distal muscles, and diminished reflexes at the ankles, biceps, triceps, and brachioradialis. ES identified a homozygous pathogenic variant in *CAPN3* (c.257del, p.(Ser86PhefsTer)), establishing the molecular diagnosis (Table S1).

A similar limb‐girdle phenotype was observed in a 15‐year‐old male proband (II:2) from Family 8, who presented with proximal muscle weakness, gait impairment, and markedly elevated CK levels. Biochemical evaluation additionally revealed severe hypertriglyceridemia. Targeted panel sequencing identified a reported homozygous splice‐region pathogenic variant in *CAPN3* (c.379+3A>G, p.(?)), suggesting calpainopathy as the underlying cause (Table S1). Two of his brothers exhibit similar disease: one (II:1) 19‐year‐old had a CK level of 8000 IU/L and received steroid therapy starting at age 12, with muscle biopsy findings consistent with muscular dystrophy; another brother (II:4), aged 8 years, had a CK level of 3000 IU/L and has been diagnosed with autism. No biological material was available for genetic testing. However, the concordant clinical features strongly indicate that both siblings are very likely affected by the same condition. Clinical and genetic findings in Families 5, 7, and 9 harboring *CAPN3* variants are summarized in Table S1.

### 3.4. *DYSF* Disrupting Biallelic Variants Cause Limb Girdle Muscular Dystrophy Recessive 2 in 15 Unrelated Families

Family 10 was recruited from Rawalpindi and belongs to the Pukhtoon ethnicity. Three female affected siblings (II:1, II:5, and II:6) were born to a consanguineous couple at term through NVD with normal birth weight and length (Figure 1), achieving all early developmental milestones. They were 34, 31, and 35 years old at examination, respectively, and presented with adolescent‐onset motor symptoms beginning between 16 and 18 years of age. Initial complaints centered around hip extensor weakness and reliance on support for ambulation. Subsequently, they developed a slow progressive pattern of weakness characterized by an inability to stand independently while retaining the ability to sit without support and walk with assistance (Table S1). None of the individuals exhibited epilepsy, and all had normal speech and preserved basic self‐care. Individual II:1 measured 153 cm in height with a HC of 52.5 cm, II:5 measured 172 cm with a HC of 53.5 cm, and II:6 measured 174 cm with a HC of 56.5 cm. ES identified a splice donor site variant NM_003494.4: c.342+1G>A, p.(?) in *DYSF* that segregated with the disease status in family (Table S1 and Figure S1). Given the close concordance between the affected individuals′ phenotype and *DYSF-*related LGMDR2, the identified pathogenic variant represents the most plausible molecular cause of the disease in this family.

Family 11, originating from southern Punjab, comprises two interconnected loops with two affected individuals in each (IV:3, IV:4 and V:1, V:7) who presented with progressive neuromuscular disease (Figure 1). The first affected individual V:1 (30 years old) experienced disease onset at 15 years of age followed by progressive muscle weakness resulting in loss of ambulation, while maintaining normal intellectual function and preserved lower limb motor function. His sister, V:7 (16 years), exhibited an abnormal gait and progressive muscular weakness. She can walk but cannot ambulate continuously for more than a few meters. The second loop includes two affected individuals, IV:3 and IV:4, who were 40 and 42 years old at the time of assessment. Individual IV:3, male, reported symptom onset at 30 years and retains limited independent walking ability. His female sibling (IV:4) demonstrates similar muscular disease who developed symptoms at 27 years of age and can ambulate only with support. However, no biological material was available for her genetic testing. Overall, the affected individuals display a shared pattern of progressive muscle weakness with variable age of onset and severity, consistent with a neuromuscular disorder segregating in this consanguineous family. Genetic analysis identified a splice‐site variant in *DYSF* (NM_003494.4): c.2163‐1G>T, p.(?), which is classified as a pathogenic variant according to the ACMG classification and is previously reported with overlapping clinical features [29] (Table S1). Sanger sequencing validated the variant that segregates in recessive pattern of inheritance (Figure S1).

In addition to the summarized features (Table S1), proband (IV:6) from Family 12 (Figure 1) initially had trouble rising from a seated position at 25 years of age. Within 2 years of onset, he was unable to lift weights. There was no history of myalgia or muscle cramps. No bulbar or sensory symptoms were noted, and L5‐S1 reflexes were absent. ES identified a previously unpublished likely pathogenic variant in *DYSF* (NM_003494.4): c.4440del, p.(Lys1480AsnfsTer6). His father, two brothers, one sister, and two aunts reportedly exhibit similar symptoms. However, no biological samples were available from extended family members for genetic testing.

In Family 17 (Figure 1), additional features of the proband (II:1) included exertional dyspnea and joint discomfort. She reported photosensitivity but no oral ulcers. Neurological examination revealed muscle tenderness and reduced ankle and knee reflexes in the lower limbs, with normal bladder and bowel function. Her obstetric history was notable for one miscarriage and one healthy daughter. Gene panel testing identified a biallelic pathogenic splice‐donor variant in *DYSF*: c.457+1G>A, providing a genetic diagnosis concordant with the clinical presentation (Table S1).

Affected female (II:2) in Family 19 exhibited a similar phenotype characterized by progressive proximal muscle weakness, waddling gait, and functional decline beginning in early adulthood (Figure 1). Muscle biopsy revealed atrophic changes, and serum aldolase levels were elevated. The patient also reported headaches, generalized body aches, dysphagia, and retrosternal pain during breathing. On examination, calf atrophy was evident without scapular winging, and proximal muscles were more severely affected than distal muscles. A younger brother was noted to be developing similar symptoms. Panel sequencing identified a homozygous nonsense variant in *DYSF* (c.1471G>T, p.(Glu491Ter)), supporting the diagnosis of dysferlinopathy (Table S1).

A more severe and progressive course was observed in Family 20 (Figure 1). The affected male (II:1) demonstrated stepwise progression of weakness from the lower to the upper limbs, leading to gait dependence and spinal postural changes, with markedly elevated CK levels. Electromyography suggested a necrotizing myopathy, and muscle biopsy revealed inflammatory changes. There were no bulbar symptoms. The patient was receiving prednisolone. A younger sister exhibited similar clinical features. Targeted sequencing identified a homozygous deletion in *DYSF* (c.2846_2852del, p.(Phe949_Val950delinsTer)), accounting for the phenotype. Clinical and genetic findings from families 13, 14, 15, 16, 18, and 21–24 are summarized (Table S1).

### 3.5. Loss‐of‐Function Variants in *LAMA2* Cause Muscular Dystrophy in Four Families

Family 25 comprises three affected siblings, IV:1 and IV:7 (male), and IV:2 (female), born to first‐cousin parents (Figure 1). Individuals were born at term following uneventful pregnancies and gestations, and were 16, 14, and 6 years old at the time of evaluation. Age of onset for the disease was noted at 6 months of age. Despite normal cognition, vision, speech articulation, and hearing, affected individuals demonstrate severe muscle weakness, congenital hypotonia, and inability to walk. Skeletal anomalies included kyphoscoliosis and quadriplegic joint contractures. They are dependent on others for feeding, self‐care, and are completely bed‐ridden. Genetic testing identified an unpublished variant in *LAMA2* (NM_000426.4): c.8596delA, p.(Ala2866LeufsTer37) as the most likely cause of the disease (Table S1 and Figure S1).

Proband (II:1) in Family 26 (Figure 1) exhibited an adult‐onset limb‐girdle pattern characterized by progressive proximal muscle weakness, waddling gait, muscle stiffness, and elevated CK levels, with myopathic features confirmed on electrophysiology and muscle biopsy. The weakness initially involved the lower limbs and was accompanied by generalized body aches, later progressing over the preceding 5–6 months to difficulty with arm abduction and activities such as brushing hair. Examination revealed a positive Gowers′ sign, asymmetric muscle involvement, and reduced bulk of the right calf, whereas facial muscles were spared. Gene Panel testing identified a homozygous frameshift variant in *LAMA2* (c.8903del, p.(Phe2968SerfsTer25)), consistent with laminin‐*α*2–related muscular dystrophy (Table S1). A positive family history was present, as an affected brother had similar symptoms with elevated CK levels and a younger sister showed comparable weakness with calf pseudohypertrophy; however, no biological samples from relatives were available for genetic testing.

Individual IV:1 from Family 27 demonstrated the earliest disease onset within the cohort, with abnormalities evident from the prenatal and neonatal periods (Figure 1). The pregnancy was notable for decreased fetal movements, although the child was delivered at term by NVD without delayed cry. He presented limb‐girdle weaknesses, hypotonia, delayed motor milestones, and persistent nonambulation. On examination during childhood, the patient exhibited an open anterior fontanelle, triangular facial appearance, right‐sided pes cavus, and chest wall deformity, whereas fundoscopy revealed no cherry‐red spots. Electrophysiological studies demonstrated a myopathic pattern. ES identified a homozygous splice‐site variant in *LAMA2* (c.3411+1G>T, p.(?)), confirming congenital‐onset laminin‐*α*2–related muscular dystrophy (Table S1). A positive family history was reported, as a maternal cousin was described to have a physical disability suggestive of a neuromuscular condition. Clinical and genetic findings in Family 28 are summarized (Table S1).

### 3.6. Biallelic Variant in *SGCA* Causes Autosomal Recessive Limb‐Girdle Muscular Dystrophy 3 in Family 29

Family 29 was recruited from the interior Sindh province and comprises four affected male individuals (V:3, V:5, V:6, V:7) from two related branches (Figure 1). All affected individuals were born at term through NVD and demonstrated a progressive neuromuscular phenotype. Individual V:3, an 8‐year‐old male, had onset of symptoms at 4 years of age, characterized by delayed developmental milestones. At his most recent evaluation, he was able to sit and stand but demonstrated an ataxic gait, marked muscle atrophy, and clinical features consistent with peripheral neuropathy. Individual V:5 was evaluated at 19 years of age. Developmental delays were noted early in life, followed by progressive decline leading to complete loss of independent standing and ambulation. He also exhibits pronounced muscle atrophy and peripheral neuropathy. His younger brother, V:6, was 12 years old at the time of assessment and showed muscle atrophy and peripheral neuropathy beginning at 8 years of age. He can sit with support but has become wheelchair dependent. Similarly, the youngest brother in this branch, V:7, was 8 years old at evaluation and had recently begun to exhibit muscle atrophy and peripheral neuropathy, accompanied by aggressive regression in motor abilities. He is now unable to stand or walk and has already lost the ability to sit independently (Table S1). ES identified a two‐base‐pair deletion in *SGCA* (NM_000023.4): c.162_163delCC, p.(Pro55ThrfsTer49), predicted to result in a frameshift and premature stop codon, likely triggering nonsense‐mediated decay of the mutant transcript. According to the ACMG criteria, this variant is classified as Pathogenic and segregates with the disease status (Table S1 and Figure S1).

## 4. Discussion

The current study reports 46 affected individuals from 29 families presenting with overlapping clinical features of muscular dystrophy and significant family histories strongly suggesting an underlying genetic cause (Figure 1). Diagnosis of LGMD based on classical paradigms typically requires invasive procedures, such as muscle biopsies and downstream histological analyses, to complement clinical data [30, 31]. These approaches impose substantial physical and healthcare burdens on patients and further strain already limited diagnostic and patient care services in LMICs. However, despite these extensive evaluations, a definitive molecular diagnosis often remains unclear. To streamline the process, avoid misdiagnosis, and reduce diagnosis waiting time, we adopted an integrative approach that combined genetic and functional investigations to identify the underlying genetic cause(s) and provide timely diagnosis to this vulnerable population.

In line with the reported literature on LGMD, variants in *CAPN3* and *DYSF* were the most common followed by *SGCA* disrupting variants [7, 32]. Among the 29 investigated families, we identified diagnostic findings in 28 families, whereas one family harbored a VUS in *COL12A1,* an emerging muscular dystrophy gene. Our experience demonstrates that an integrated diagnostic approach to LGMD is highly effective, enabling a molecular diagnosis in 46 individuals. We therefore propose to incorporate genetic investigations in LMICs as a synergistic component to facilitate clinically meaningful outcomes in a timely manner.

Family 1 harbors a variant in *COL12A1.* Mutations in this gene cause Ullrich congenital muscular dystrophy 2 (UCMD2; MIM# 616470) in recessive and Bethlem myopathy 2 (BTHLM2; MIM# 616471) in dominant pattern of inheritance. Recessive disease is marked by congenital hypotonia, proximal muscle weakness affecting the pelvic and shoulder girdles, hyperlaxity, contractures, spinal deformities (kyphosis/scoliosis), and typically preserved cognition [33, 34], whereas dominant heterozygous variants demonstrate milder presentations [35]. The clinical presentation of affected individuals (IV:2, IV:4) in Family 1 (Table S1) overlaps with the reported features of UCMD2. However, respiratory involvement and feeding difficulties, which have been described in UCMD2 cases harboring biallelic or compound heterozygous predicted loss‐of‐function variants (at least one allele) [36, 37], were not observed. Since the variant identified in our study is a missense change, differences in functional impact may underlie the milder clinical presentation. This is consistent with other reported UCMD2 cases carrying missense variants that do not exhibit respiratory or feeding involvement [34, 38, 39] emphasizing the need for further functional and comparative studies.

The identified missense variant in Family 1, affecting *COL12A1* (NM_004370.6): c.3151G>C, p.(Val1051Leu), was associated with reduced transcript abundance in patient‐derived fibroblasts (Figure 3), suggesting that, despite being a coding missense variant, it may influence transcript stability or processing. The underlying mechanism responsible for this reduction could not be established in the present study. Further studies will be required to determine whether the reduced transcript levels result from transcript degradation, aberrant splicing, or another regulatory mechanism.

*CAPN3* dysfunction is known to cause LGMD2A/LGMDR1 (MIM# 253600) in recessive pattern presenting with symmetric proximal limb‐girdle weakness, scapular winging, flexion contractures, and elevated CK, generally starting in adolescence/young adulthood [40]. We identified pathogenic *CAPN3* variants in eight families (Table S1). The clinical presentation of the affected individuals is consistent with the established manifestations of LGMDR1, most prominently the predominant adolescent onset and loss of ambulation later in adulthood [40, 41]. However, the presence of seizures in Family 2‐II:3 and early disease onset in Family 7 proband is atypical for pure calpainopathy (Table S1). In Families 3 and 4, the observation of early severe distal muscle involvement and the distal bias of muscular weakness (Table S1) extend known phenotype boundaries for recessive CAPN3 defects [40]. Overall, our clinical findings are in agreement with the reported recessive calpainopathy manifestations, and we expanded the mutation spectrum of LGMD2A/LGMDR1.

We observed likely founder effects in our cohort. The identification of *CAPN3*: c.1469delinsTC, p.(Arg490LeufsTer87) variant in three apparently unrelated families (Families 2–4), as well as *CAPN3*: c.379+3A>G, p.(?) in two additional families (Families 8–9) originating from geographically distant regions of Pakistan, strongly suggests a shared ancestral origin. Notably, Arg490 appears to be a mutational hotspot, as p.(Arg490Gln) and p.(Arg490Trp) have both been previously established as pathogenic missense variants. Similarly, *DYSF*: c.5341‐2A>C, p.(?) was identified in three independent families (Families 14, 15, and 16), further supporting this notion. This phenomenon is well documented in the Pakistani population [24] and is facilitated by long‐standing sociocultural practices including endogamy and consanguineous marriages, which leads to the variant enrichment and homozygosity [42, 43]. These observations emphasize the urgent need for public awareness, premarital and carrier screening, and community‐sensitive genetic counseling to mitigate further genetic burden and prevent long‐term population‐level health consequences.

In our cohort, 15 families (Families 10–24) carry biallelic pathogenic/likely pathogenic variants in *DYSF,* including unique splice‐site (*n* = 5), frameshift (*n* = 4), missense and nonsense (*n* = 2 each) variants. Among these 13 unique variants, eight are being reported for the first time in current study (Table S1). *DYSF* disrupting variants cause a clinically heterogeneous group of autosomal recessive muscular dystrophies, called dysferlinopathies [44], including LGMDR2/LGMD2B (MIM# 253601) which predominantly affects proximal muscles; Miyoshi muscular dystrophy 1 (MMD1; MIM# 254130), characterized by distal posterior leg involvement; and distal myopathy with anterior tibial onset (DMAT; MIM# 606768). The hallmark clinical features include adolescent/young‐adult onset, progressive weakness, marked CK elevation, and defective sarcolemma repair [45, 46]. The absence of overt calf myalgia and distal muscle involvement in our cohort makes MMD1 unlikely and instead supports a pelvic‐girdle predominant phenotype within the dysferlinopathy spectrum, consistent with LGMDR2. The shared clinical features and pathogenic *DYSF* variants identified in our cohort further strengthen the causal association with LGMDR2 and expand the mutational spectrum of the gene.

*LAMA2* encodes the alpha‐2 subunit of laminin, a protein that maintains assembly and integrity of the basement membrane in skeletal muscle cells, Schwann cells, astrocytes, and pericytes [47]. *LAMA2* dysfunction leads to two variable manifestations of recessive muscular dystrophy ranging from a severe early onset congenital merosin‐deficient muscular dystrophy (MDC1A; MIM# 607855) to a mild late‐onset limb‐girdle muscular dystrophy 23 (LGMDR23; MIM# 618138). Along with the common clinical features of *LAMA2-*related dystrophies, Families 25, 27, and 28 exhibited infantile onset hypotonia, delayed motor milestones, and a severe clinical course (Table S1). The congenital presentation and early functional impairment strongly indicate a more severe, likely near‐complete laminin‐*α*2 deficiency consistent with MDC1A [48]. In contrast, the proband in Family 26 is best classified as the case of LGMDR23 given the late disease onset (24 years), progressive proximal limb‐girdle weakness with preserved early development supporting a milder partial merosin‐deficient presentation.

Family 29 in our cohort harbors a biallelic frameshifting variant in *SGCA* (*α*‐sarcoglycan) which is a component of the sarcoglycan subcomplex within the dystrophin‐glycoprotein complex (DGC) that stabilizes the sarcolemma during muscle contraction/relaxation; disruption leads to myofiber necrosis and fibrosis [49, 50]. *SGCA* dysfunction is known to cause LGMDR3/LGMD2D (MIM# 608099) with childhood onset, progressive proximal weakness, calf pseudohypertrophy, waddling gait, and high CK [51]. The four affected males in Family 29 exhibited early motor regression, marked proximal atrophy, peripheral neuropathy features, and loss of independent ambulation in the teens (Table S1), which aligns with the LGMDR3 phenotype [52].

In conclusion, we diagnosed 29 families comprising 46 affected individuals with LGMD, highlighting substantial clinical and genetic heterogeneity. We identified 17 unreported variants in *COL12A1*, *CAPN3*, *DYSF*, *LAMA2,* and *SGCA* expanding the mutation spectra of these genes. Our findings support the incorporation of genetic testing as a first‐tier diagnostic approach, particularly in LMICs settings, where it can improve diagnostic accuracy, shorten the diagnostic odyssey, and reduce dependence on invasive and resource‐intensive procedures. In addition, community‐based recruitment for genetic diagnosis appears to be more efficient for extended family carrier screening and alleviating the burden on limited healthcare resources. Furthermore, our findings highlight the urgent need to improve public literacy on implications of consanguinity.

## Author Contributions

Conceptualization: Hammad Yousaf, Zafar Iqbal, and Ambrin Fatima. Research subjects′ provision: Hammad Yousaf, Lubaba Bintee Khalid, Affan Ahmed, Rafia Zafar Ghumman, Shaheer Hassan, Hijab Zahra, Ghazala Zafar, Kinza Rubab, Hazaratullah Azakai, Haq Nawaz Khan, Qamar Mahmood, Asmat Ali, Bilal Ahmad Mian, Zafar Ali, Mohammad Raza, Sanam Bano, Khairunnisa Mukhtiar, Uzma Abdullah, Shahnaz H. Ibrahim, Shahid M. Baig, Mathias Toft, Salman Kirmani, Zafar Iqbal, and Ambrin Fatima. Clinical evaluation: Mohammad Raza, Sanam Bano, Khairunnisa Mukhtiar, Shahnaz H. Ibrahim, and Salman Kirmani. Experimental investigation: Hammad Yousaf, Lubaba Bintee Khalid, Rafia Zafar Ghumman, Shaheer Hassan, Hijab Zahra, Ghazala Zafar, Haq Nawaz Khan, Asmat Ali, Bilal Ahmad Mian, Zafar Ali, Uzma Abdullah, Shahid M. Baig, Mathias Toft, Zafar Iqbal, and Ambrin Fatima. Writing—original draft: Hammad Yousaf, Rafia Zafar Ghumman, and Shaheer Hassan. Writing—review and editing: Hammad Yousaf, Zafar Iqbal, and Ambrin Fatima. Supervision: Zafar Iqbal and Ambrin Fatima. Zafar Iqbal and Ambrin Fatima should be regarded as co‐corresponding authors.

## Funding

This work was supported by institutional funds from the Aga Khan University awarded to Ambrin Fatima.

## Disclosure

All authors read and approved the manuscript.

## Ethics Statement

This study was approved by the institutional review board of the Aga Khan university vide letter no. 2021‐65141‐9346 and is conducted in line with the principles of Declaration of Helsinki. Informed consent was obtained from all participants or their legal guardians for participation in the study and the publication of clinical information.

## Conflicts of Interest

The authors declare no conflicts of interest.

## Supporting Information

Additional supporting information can be found online in the Supporting Information section.

## Supporting information


**Supporting Information 1** Figure S1: Sanger sequencing chromatograms for the multiplex families with additional individuals available for testing.

**Supporting Information 2** Table S1: Summary of the investigated individuals and identified variants in the current study.

## Data Availability

The data that support the findings of this study are available in the Supporting Information of this article.
