# Supplementary material for: Integrated Approach to Diagnosing Limb‐Girdle Muscular Dystrophies in Resource‐Limited Settings
Source: Hum Mutat. 2026 Oct 2;2026:1321391. doi: 10.1155/humu/1321391 (PMC13633453; doi:10.1155/humu/1321391)
Supplement: Supplementary file 1 [file HUMU-2026-1321391-s002.pdf]

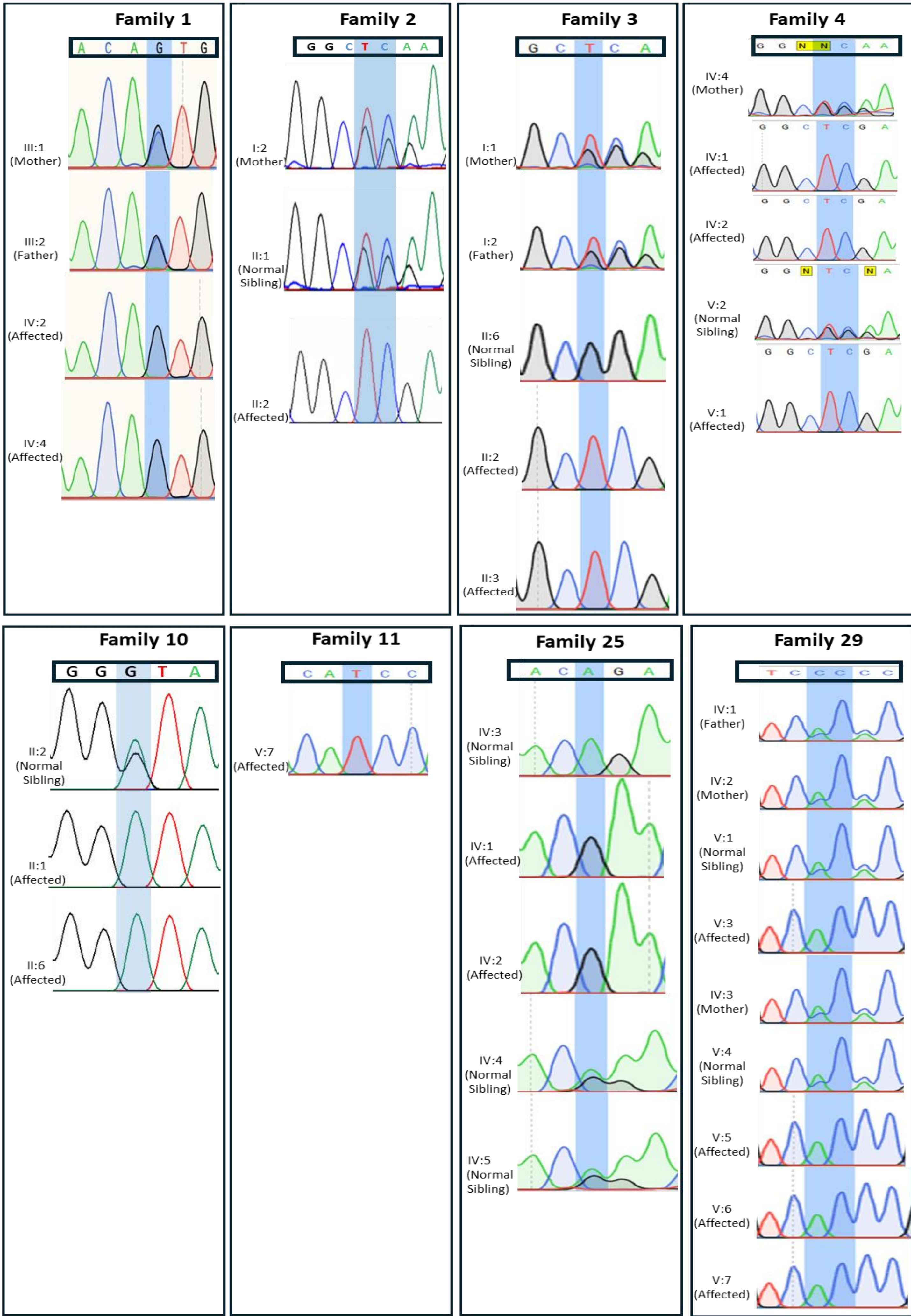

Family 1
A C A G T G
III:1 (Mother)
III:2 (Father)
IV:2 (Affected)
IV:4 (Affected)
Family 2
G G C T C A A
I:2 (Mother)
II:1 (Normal Sibling)
II:2 (Affected)
Family 3
G C T C A
I:1 (Mother)
I:2 (Father)
II:6 (Normal Sibling)
II:2 (Affected)
II:3 (Affected)
Family 4
G G N N C A A
IV:4 (Mother)
G G C T C G A
IV:1 (Affected)
G G C T C G A
IV:2 (Affected)
G G N T C N A
V:2 (Normal Sibling)
G G C T C G A
V:1 (Affected)
Family 10
G G G T A
II:2 (Normal Sibling)
II:1 (Affected)
II:6 (Affected)
Family 11
C A T C C
V:7 (Affected)
Family 25
A C A G A
IV:3 (Normal Sibling)
IV:1 (Affected)
IV:2 (Affected)
IV:4 (Normal Sibling)
IV:5 (Normal Sibling)
Family 29
T C C C C C
IV:1 (Father)
IV:2 (Mother)
V:1 (Normal Sibling)
V:3 (Affected)
IV:3 (Mother)
V:4 (Normal Sibling)
V:5 (Affected)
V:6 (Affected)
V:7 (Affected)
